# Impaired Oocyte Quality Induced by Dehydroepiandrosterone Is Partially Rescued by Metformin Treatment

**DOI:** 10.1371/journal.pone.0122370

**Published:** 2015-03-26

**Authors:** Ying Huang, Yang Yu, Jiangman Gao, Rong Li, Chunmei Zhang, Hongcui Zhao, Yue Zhao, Jie Qiao

**Affiliations:** 1 Reproductive Medical Center, Department of Obstetrics and Gynecology, Peking University Third Hospital, Beijing, China; 2 Key Laboratory of Assisted Reproduction, Ministry of Education, Beijing, China; 3 Beijing Key Laboratory of Reproductive Endocrinology and Assisted Reproductive Technology, Beijing, China; Institute of Zoology, Chinese Academy of Sciences, CHINA

## Abstract

The present study evaluated the influence of hyperandrogenism on oocyte quality using a murine PCOS model induced by dehydroepiandrosterone (DHEA) and further explored the effect of metformin treatment. Female BALB/c mice were treated with a vehicle control or DHEA (6 mg /100 g body weight) or DHEA plus metformin (50 mg /100 g body weight) for 20 consecutive days. DHEA-induced mice resembled some characters of human PCOS, such as irregular sexual cycles and polycystic ovaries. After the model validation was completed, metaphase II (MII) oocytes were retrieved and subsequent analyses of oocyte quality were performed. DHEA-treated mice yielded fewer MII oocytes, which displayed decreased mtDNA copy number, ATP content, inner mitochondrial membrane potential, excessive oxidative stress and impaired embryo development competence compared with those in control mice. Metformin treatment partially attenuated those damages, as evidenced by the increased fertilization and blastocyst rate, ATP content, GSH concentration and GSH/GSSG ratio, and decreased reactive oxygen species levels. No significant difference in normal spindle assembly was observed among the three groups. During in vitro maturation (IVM), the periods of germinal vesicle breakdown (GVBD) and the first polar body (PB1) extrusion were extended and the maturation rate of GVBD oocytes was decreased in DHEA mice compared with controls. Metformin treatment decreased the time elapsed of GVBD while had no effect on PB1 extrusion. These results indicated that excessive androgen is detrimental to oocyte quality while metformin treatment is, directly or indirectly, beneficial for oocyte quality improvement.

## Introduction

Polycystic ovary syndrome (PCOS) is the most common female endocrine disorder and has a cluster of characteristics, such as oligo-ovulation or anovulation, hyperandrogenism and multicystic ovaries. Metabolic abnormalities, including obesity, insulin resistance, type 2 diabetes and cardiovascular disease are also found in PCOS. Further, PCOS is one of the main reasons for female infertility, affecting 5–10% women of reproductive age. During in vitro fertilization (IVF) treatment, although PCOS patients obtain increased oocytes, they often have lower rates of fertilization, cleavage and implantation and a higher rate of miscarriage, which might be associated with poor quality of the oocytes and the resulting embryos[1]. Hyperandrogenism, a principal characteristic of PCOS, is involved in the occurrence and development of this disease. It has been confirmed that the majority of PCOS patients (60–80%) have biochemical and/or clinical hyperandrogenism[2]. Elevated androgen levels have been reported to be detrimental to IVF outcomes and decrease the oocyte maturation rate during in vitro maturation (IVM)[3,4], suggesting that hyperandrogenism may hinder the maturation and development potential of oocytes. However, the direct evidence is still absent.

In addition to the fecundity effect, hyperandrogenism can also prompt insulin resistance and induce metabolic disturbances in PCOS patients [5,6]. Metformin is a widely used oral hypoglycemic agent in the treatment of type 2 diabetes, metabolic syndrome and PCOS patients. Mechanically, metformin induces an increase in muscle glucose uptake and a decrease in hepatic glucose production by enhancing insulin sensitivity. Although the efficacy of metformin on systemic metabolism improvement in PCOS patients has been confirmed, its effect on reproduction remains controversial. Some investigators showed that metformin does help to restore the menstrual cycle and fertility, and improve conception rates following therapies such as ovulation induction and controlled ovarian hyperstimulation prior to IVF[7,8,9]. In contrast, other studies demonstrated that metformin does not offer any clinical benefit in terms of ovulation rate or pregnancy outcome[10,11,12].

Recently, a study by Palomba S et al. demonstrated that metformin reduced the intrafollicular androgen level and improved ovarian insulin resistance and ovary morphology in PCOS patients, suggesting a local benefit on the ovary[13]. In addition, hyperandrogenic anovulatory mutant murine models treated with metformin achieved a greater number of mature oocytes and overall oocytes compared to untreated controls[14]. However, the assessment of the resulting oocyte quality is absent. In this study, we evaluated the influence of hyperandrogenism on oocyte quality in vivo using a murine PCOS model induced by DHEA and further explored the effect of metformin treatment.

## Materials and Methods

All of the chemicals used in this study were purchased from Sigma-Aldrich Chemical Co. (St. Louis, MO, USA) unless otherwise stated.

### Ethical approval

All procedures involving mice were operated under strict criteria on the basis of the Guide for Care and Use of Laboratory Animals of Peking University, and the protocol was approved by the Institutional Animal Care and Use Committee of Peking University Third Hospital.

### PCOS model

Female prepuberal (25 days old) mice of the BALB/c strain (Vital River Laboratories, Beijing, China) were randomly divided into three groups (control group, DHEA group, DHEA+metformin group). Animals of the DHEA group were injected daily with DHEA (6 mg/100 g body weight, dissolved in 0.05 ml sesame oil) and given saline (0.2 ml) orally with a cannula for 20 consecutive days. The DHEA+metformin group animals were injected daily with DHEA and given 1,1-dimethylbiguanide hydrochloride (metformin, 50 mg/100 g body weight in 0.2 ml saline) orally for 20 days. The control group animals were administered oil (0.05 ml) and saline (0.2 ml). All of these mice were raised and housed in the Animal Center of the Medical College of Peking University according to the national legislation for animal care. All mice were maintained under controlled temperature and lighting conditions and allowed free access to food and water. Throughout the whole treatment period, the animals were weighed every two days, and vaginal smears were taken daily beginning 10 days after the first injection up to the end of experiments. The stage of cyclicity was determined by microscopic analysis of the predominant cell type in vaginal smears. After 20 days of treatment, eight to ten mice per group were assessed whether the model was successfully established. As all mice from DHEA and DHEA+metformin group were completely acyclic and remained in constant estrus while control mice had normal cycle. To eliminate the effect of estrous cycle on other detecting indicators, only those mice that sacrificed on the estrus stage in the control group were used for model validation. Magnetic resonance imaging (MRI) and oral glucose tolerance tests (OGTT) were performed before the mice were anaesthetized with ether and euthanized by cervical dislocation. The rest of the animals were used for oocyte collection and the following quality evaluation. 7–8 weeks old mice were used in all experiments, with the exception of the body weight and the estrous cycle evaluation. During oocyte quality evaluation, 3–5mice per group was used in each experiment except for 6–10 mice per group was used in *in vitro* fertilization and *in vitro* maturation.

### Body composition analysis

To determine body fat mass composition, mice were placed in a clear plastic holder without anesthesia or sedation and inserted into the EchoMRI TM device (Echo Medical Systems, USA) at the end of the treatment.

### OGTT

Mice were fasted for 8 hours before the OGTT experiment. Glucose levels were measured by tail vein blood sampling using a blood glucose meter (Sinocare Inc., Changsha, China). After the fasting, the glucose levels were measured, then the mice were administered glucose (2 g/kg body weight) by oral gavage, and tail sampling was performed at the time point of 30, 60, 90and 120 minutes.

### Blood sampling for testosterone, insulin and lipid profiles

At the end of the treatment, blood was drawn from the inner canthus after the mice were fasted for 8 hours. The serum was immediately separated and kept in -80°C for subsequent hormone determinations. Testosterone and fasting insulin levels were measured with radioimmunoassay kits (Beijing North Institute of Biological Technology, Beijing, China). Lipid profiles were determined with biochemical analysis kits (China Diagnostics Medical Corporation, Beijing, China).

### Ovarian morphology

Immediately after the collection of blood samples, the ovaries were quickly removed and fixed in 4% paraformaldehyde. After embedding in paraffin and following routine histological procedures, 5-μm sections were mounted on glass slides and stained with hematoxylin and eosin for observation under the light microscopy.

### Oocyte collection

To collect MII oocytes, mice from the control, DHEA and DHEA+metformin groups were superovulated by the intraperitoneal injection of 10 IU of equine chronic gonadotropin (Hua Fu Biotechnology Company, Tianjin, China), followed by 10 IU of human chorionic gonadotropin (Hua Fu Biotechnology Company, Tianjin, China) 48 h later. Cumulus-oocyte complexes (COCs) were collected from oviductal ampullae 14–16 h after hCG injection, and the cumulus cells were removed by brief incubation in 0.2% hyaluronidase. Denuded oocytes were observed under stereomicroscopy. Oocytes with a round clear zona pellucida, a small perivitelline space and a pale, moderately granular cytoplasm that does not contain inclusions were considered to be “normal” [15], and only these “normal” oocytes were used for further research.

To collect GV oocytes, ovaries were obtained from mice that were not stimulated by any exogenous hormone. The collected ovaries were dissected by manual rupturing in G-MOPS (Vitrolife, Gothenburg, Sweden), and the separated GV oocytes were collected for in vitro maturation (IVM).

### In vitro fertilization and embryo culture

The epididymal spermatozoa were retrieved from the cauda epididymis of 8–10 week-old BALB/c male mice, and the sperm suspensions were capacitated in human tubal fluid (HTF)/10% serum protein substitute (SPS) medium for 90 min at 37°C in 5% CO_2_ and 95% humidity. Ovulated oocytes with intact cumulus masses were recovered from oviducts and maintained in HTF. Then, the capacitated sperm in a concentration of 1–2×10^6^/ml was added to the COCs droplet and cultured at 37°C, 5% CO_2_ and 95% humidity. Six hours later, the oocytes were removed and examined for fertilization. Fertilization was assessed by the presence of two pronuclei (2PN). Twenty to thirty fertilized embryos were transferred to one KSOM (K Simplex Optimization medium) droplet with a 30ul volume at 37°C, 5% CO_2_ and 95% humidity. Fertilized embryos were cultured to the blastocyst stage for 4 days, and the development was recorded every 24 h.

### In vitro maturation

Immature oocytes were cultured in an IVM medium (LifeGlobal, Guilford, CT, USA) for 24h in a humid atmosphere at 37°C with 5% CO_2_ in a live cell station (PerkinElmer, Massachusetts, USA). Twenty denuded GV oocytes were cultured in a 20-μl drop of IVM medium, and pictures were taken every 30 seconds. The beginning time and the time of germinal vesicle breakdown (GVBD) and first polar body (PB1) extrusion were recorded, and the time elapsed until GVBD and PB1 extrusion was calculated. After 24 hours, mature oocytes with PB1 extrusion were collected for spindle and chromosome staining.

### Immunofluorescence

For spindle and chromosome analysis, denuded MII oocytes were placed in 4% paraformaldehyde for 30 min after rinsing twice in PBS. Then the oocytes were permeabilized with 1% Triton X-100 and incubated overnight at 4°C. The next day, the permeabilized oocytes were incubated with PBS supplemented with 1% bovine serum albumin (BSA) for 1–2 hours at room temperature. Subsequently, the oocytes were transferred to a droplet containing monoclonal anti-alpha-tubulin-FITC antibody (1:100 dilution in BSA) for 1–2 hours. After three washes, the oocytes were stained with 10 μg/ml of propidium iodide for 5–10 min. Finally, the oocytes were mounted on glass slides and examined with a confocal laser scanning microscope (LSM710 Carl Zeiss, Oberkochen, Germany).

For inner mitochondrial membrane potential or reactive oxygen species (ROS) staining, denuded MII oocytes were incubated in PBS containing 2 μM JC-1 fluorochrome (Invitrogen, Carlsbad, CA) or 10 μM DCFH-DA(Nanjing Jiancheng Bioengineering Institute, Nanjing, China) at 37°C, 5% CO2 in air for 30 min. Meanwhile, the negative controls were cultured in PBS for 30min. After washing 3 times with PBS supplemented with BSA, oocytes were placed in glass bottom cell culture dishes (NEST Biotechnology Co.LTD, Wuxi, China) and analyzed by fluorescence microscopy. The fluorescence intensity was measured with Image J software.

For lipid droplet staining, denuded MII oocytes were placed in 4% paraformaldehyde for 45 min, washed three times with PBS and then stained with 1 μg/ml BODIPY 493/503 (Invitrogen) lipophilic dye for 1 h in the dark at room temperature. Meanwhile, the negative control oocyte was cultured in PBS for 1 h. After staining, oocytes were washed 3 times in PBS supplemented with BSA and placed on a confocal dish. Images were taken by confocal microscopy at × 20 magnification.

### Measurement of GSH and GSSG content

For reduced glutathione(GSH) and oxidized disulfide (GSSG) content evaluation, a commercial assay kit (Beyotime Institute of Biotechnology, Shanghai, China) was used. Procedures were performed according to the manufacturer’s instructions and a previous literature [16]. A total of 20–40 MII oocytes from each group were mixed for detection at a time. Using liquid nitrogen and 37°C water, the mixture was rapidly frozen and thawed twice. Then the mixture was put on the ice for 5 min and centrifuged at 10,000 g at 4°C for 10 min. The supernatant was collected for total glutathione detection. For the GSSG test, GSH was removed by diethyl maleimide, 2-vinylpyridine. By subtracting the GSSG content from the T-GSH content, the content of GSH can be determined.

### Measurement of ATP content

For the measurement of the ATP content in MII oocytes, a commercial ATP assay kit (Beyotime Institute of Biotechnology, Shanghai, China) was used and the detection was performed with a luminometer (Bioluminat Junior; Berthold, Wildbad, Germany). 20–40MII oocytes from each group were vortexed in 100 μl schizolysis solution for 1 minute for lysis. After that, the mixture was centrifuged at 12,000 g for 10 min at 4°C, and the supernatant was collected and stored at -80°C until further use. Eight standard points (0, 0.01, 0.05, 0.1, 0.5, 1.0, 2.5 and 3.0 μmol/L of ATP) were performed in each assay, with the formula derived from the standard curve, the ATP content was calculated.

### Mitochondrial DNA copy number quantification

One single MII oocyte was added to a PCR tube containing 10 μl lysis buffer (50mM Tris-HCL, 0.1mM EDTA, 0.5%Tween-20 and 200μg/ml Proteinase K). Then the tube was incubated at 55°C for 2 h and 95°C for 10 min. After that, the samples were used for real-time qPCR detection directly by an ABI 7500 real-time PCR system (Applied Biosystems, Forest City, CA). The procedure to obtain the purified plasmid standard DNA used to produce a standard curve and the mitochondrial DNA (mtDNA) quantitative real-time PCR were performed as previously described[16]. All of the reactions were run in duplicate, and the mean quantity of mtDNA was calculated using the 7500 ABI system software based on the standard curve.

### Statistical analysis

Data are represented as the mean± SEM from at least three replicates per experiment. Statistical significance was determined as indicated; by one-way analysis of variance followed by Bonferroni, Dunnett or Scheffe post hoc analysis, chi square tests, as appropriate using SPSS 16.0 software. A P value of<0.05 was considered statistically significant.

## Results

### DHEA disrupted estrous cyclicity and ovarian morphology in mice

**Fig 1:**
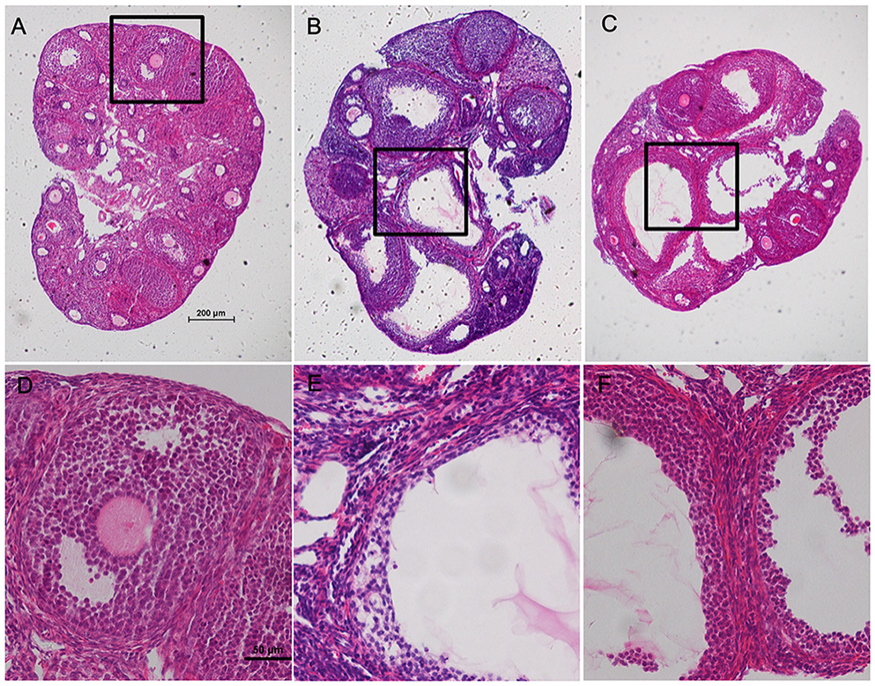
Ovary morphology of the control, DHEA and DHEA+metformin mice. Histologic sections (hematoxylin-eosin staining) of representative ovaries on day 25 of treatment. A, Ovary from a normal cycling control mouse, showing corpus lutea and follicles at different stages. B, Ovary from a DHEA-treated mouse, showing many cystic follicles. C, Ovary from a DHEA+metformin-treated mouse, showing multiple cystic follicles. D, Higher magnification of the boxed area in A, showing a normal antral follicle. E, Higher magnification of the boxed area in B. F, Higher magnification of the boxed area in C, showing cystic follicle.

To evaluate the reproductive function of the mice, estrous cycle and ovarian morphology were assessed. All control mice had a normal estrous cycle of 4–5 days. However, mice treated with DHEA were completely acyclic and remained in constant estrus. Ovaries from control mice exhibited follicles in different stages of development. Neither structural abnormalities nor ovarian cysts were found in these ovaries (Fig 1A). However, the ovaries from DHEA treated mice were obviously swelled and had multiple cystic follicles (Fig 1B and 1E). Unexpectedly, metformin administration did not improve estrous cyclicity and ovarian morphology (Fig 1C and 1F).

### Metabolic disorder induced by DHEA was partially rescued by metformin

**Fig 2:**
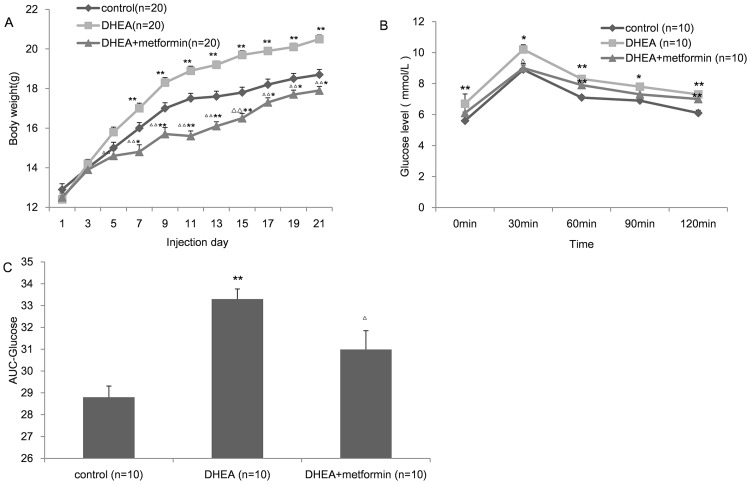
Effect of DHEA and metformin on maternal weight and glucose tolerance. A, Growth curves of mice exposed to vehicles (control), DHEA, or DHEA+metformin (from day 1 to day 21). Body weight was monitored every two days. The absence of a superscript in the column indicated that there was no significant difference in these three groups. B, Plasma glucose levels during the oral glucose tolerance test (OGTT). C, Area under the curve (AUC) for glucose in the mice. Values are the mean±SEM, n shows the number of mice, * P<0.05, ** P<0.01 versus control; Δ P<0.05, Δ Δ P<0.01 versus DHEA group by one-way ANOVA, Bonferroni post test.

At the end of the model establishment, the weight gain and fat mass of the DHEA-treated mice were significantly higher than those of the age-matched controls. However, when metformin was administered simultaneously, body weight and fat mass were significantly decreased (Table 1, Fig 2). Besides, DHEA-treated mice showed impaired glucose tolerance, increased serum TG and CHO levels, while metformin administration partially reversed these abnormities. However, the fasting serum insulin level had no difference among the three groups. The details are shown in Table 1 and Fig 2. In addition, the testosterone level was dramatically increased after DHEA injection, whereas metformin decreased it to a certain degree (Table 1).

**Table 1:** Effect of DHEA and metformin on maternal metabolic parameters and serum hormone levels.

	Control (n = 8)	DHEA (n = 8)	DHEA+metformin (n = 8)
Body fat (g)	1.74±0.11	2.13±0.029**	1.5±0.074^ΔΔ^
Body fat (% of BW)	9.05±0.55	9.98±0.23	7.84±0.36^ΔΔ^
Fasting insulin(uIU/ml)	11.88±0.31	13.49±0.74	12.89±0.91
Serum TG(mmol/L)	1.32±0.03	1.94±0.06**	1.67±0.12* ^Δ^
Serum CHO(mmol/L)	2.94±0.07	3.90±0.06**	3.18±0.08^ΔΔ^
Serum HDL(mmol/L)	1.58±0.07	1.37±0.10	1.58±0.09
Serum LDL(mmol/L)	0.185±0.0066	0.194±0.0089	0.175±0.0118
Testosterone(ng/ml)	0.038±0.008	5.068±1.15**	3.647±0.76** ^Δ^

BW, Body weight. Values are the mean±SEM,

* P<0.05,

** P<0.01 versus control;

^Δ^ P<0.05,

^ΔΔ^ P<0.01 versus DHEA group by one-way ANOVA, Bonferroni or Dunnett post test.

### DHEA induced ovulatory dysfunction during superovulation, which was partially reversed by metformin treatment

To investigate the influence of DHEA on ovulatory function during superovulation, mice were primed with PMSG and hCG. As shown in Table 2, DHEA treatment significantly decreased the recovered oocyte number per mouse. Under stereomicroscopy, two different stages of oocytes were obseved, i.e. metaphase I (MI) oocytes with granulosa cells closely enclosed, and metaphase II (MII) oocytes. Among the MII oocytes, enlarged perivitelline space, fragmented cytoplasm or giant polar bodies were considered to be morphological abnormalities. DHEA administration significantly increased the rate of oocyte stayed at MI and decreased the rate of normal MII oocytes when compared to the controls. Although metformin treatment had no effect on the oocyte retrieval number, it increased the percentage of normal MII oocytes. The details are shown in Table 2.

**Table 2:** Effect of DHEA and metformin on ovulatory function.

	Control (n = 18)	DHEA (n = 20)	DHEA+metformin (n = 20)
No. of total oocytes	373	229	204
No. of oocytes from per mouse	20.21±3.37	11.39±1.91*	10.20±0.69*
Percentage of normal MII oocytes (%)	85.02±0.44	71.65±2.88**	81.49±2.16^Δ^
Percentage of abnormal MII oocytes (%)	11.39±1.43	14.76±2.79	12.28±2.08
Percentage of MI oocytes (%)	3.58±1.70	11.88±2.46*	6.23±1.40

MI, metaphase I; MII, metaphase II. Values are the mean±SEM,

* P<0.05,

** P<0.01 versus control;

^Δ^ P<0.05 versus DHEA group by one-way ANOVA, Scheffe post test.

### Compromised oocyte and embryo development potential induced by DHEA was rescued by metformin treatment

To confirm the effects of DHEA and metformin on the development competence of oocytes, the development of fertilized embryos were analyzed. Compared with controls, DHEA treatment significantly decreased the fertilization ability of oocytes and the 2-cell, 8-cell and blastocyst formation rates. As shown in Table 3, when metformin was administered simultaneously, fertilization rate and blastocyst rate were greatly increased, with a comparable level to the controls. Although the 2-cell rate and 8-cell rate were also increased after metformin treatment, there is no significant difference between the DHEA and DHEA+metformin group.

**Table 3:** Effect of DHEA and metformin on the development potential of oocytes and the resulting embryos.

	Control (n = 10)	DHEA (n = 10)	DHEA+metformin (n = 10)
No. of total oocytes	219	119	115
Fertilization rate (%)	82.98±2.74	61.27±3.28**	79.66±2.77^Δ Δ^
2-cell rate (%)	78.55±2.81	47.71±3.57**	62.75±5.47*
8-cell rate (%)	60.48±2.20	37.64±1.56**	48.48±3.85*
Blastocyst rate (%)	49.19±2.71	31.29±2.17**	40.55±2.23^Δ^

Values are the mean±SEM,

* P<0.05,

** P<0.01 versus control;

^Δ^ P<0.05,

^Δ Δ^ P<0.01 versus DHEA group by one-way ANOVA, Bonferroni post test.

### The redundant oocyte lipid content induced by DHEA in MII oocytes was reversed by metformin treatment

**Fig 3:**
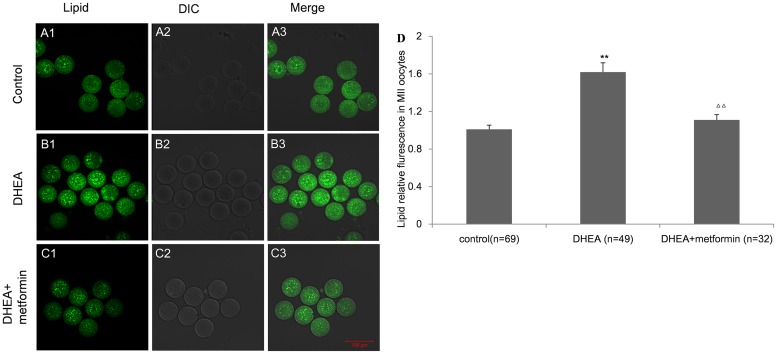
Effect of DHEA and metformin on the lipid content in MII oocytes. Figure A-C show representative images of the lipid content in the control (FigureA1-3), DHEA (FigureB1-3) and DHEA+metformin group (Figure C1-3) (Bar is 100μm). D, The BODIPY fluorescence intensity of a single MII oocyte. Data are the mean±SEM; n shows the number of oocytes from 3–5mice per group. ** P<0.01 versus control; Δ Δ P<0.01 versus DHEA group by one-way ANOVA, Dunnett post test.

As maternal lipid metabolism was greatly changed by DHEA, we wondered whether the lipid content of oocytes was also influenced. MII oocytes from DHEA mice showed visibly higher levels of lipid than those from the controls (1.62±0.099 vs. 1.01±0.044), whereas metformin treatment (1.11±0.056) completely reversed the lipid augmentation in DHEA mice, which showed the similar lipid content to the controls (Fig 3).

### DHEA induced mitochondrial dysfunction in MII oocytes, which was partially rescued by metformin treatment

**Fig 4:**
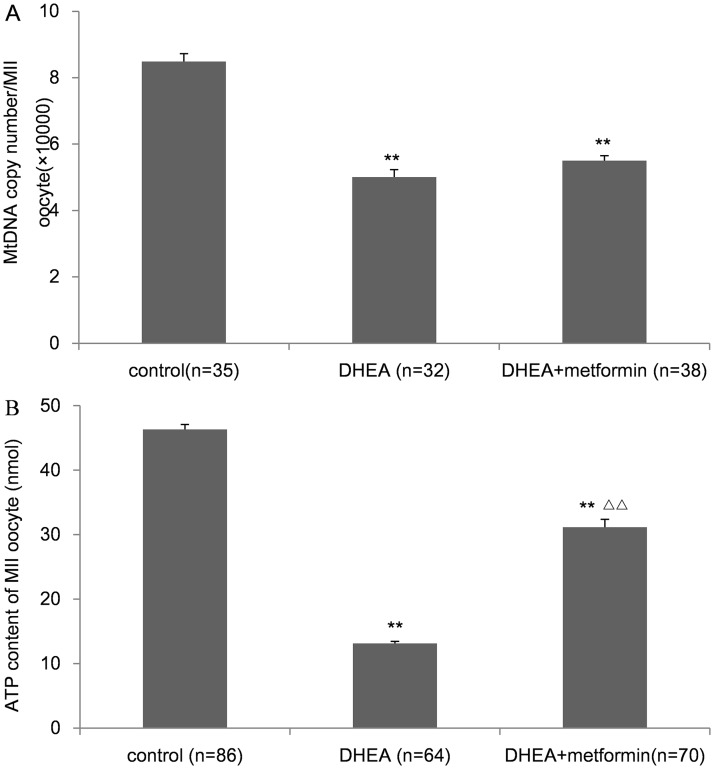
Mitochondrial function of MII oocytes in control, DHEA and DHEA+metformin group. A, MtDNA copy number per MII oocyte. B, ATP content per MII oocyte. Data are the mean±SEM; n shows the number of oocytes from 3–5mice per group. ** P<0.01 versus control; Δ Δ P<0.01 versus DHEA group by one-way ANOVA, Scheffe or Bonferroni post test.

The mitochondrion is the primary organelle of metabolism in cells. To evaluate mitochondrial function in MII oocytes, the mtDNA copy number and ATP content were assessed. The average mtDNA copy number (Fig 4A) and ATP content (Fig 4B) per MII oocyte in the DHEA group was significantly lower than those in the control group. Although metformin treatment did not modify the mtDNA copy number, the ATP content was much increased (Fig 4B).

### DHEA decreased the inner mitochondrial membrane potential in MII oocytes

**Fig 5:**
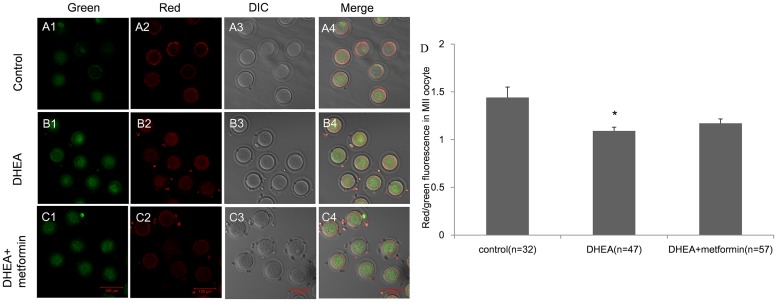
Mitochondrial membrane potential (MMP) of MII oocytes in control, DHEA and DHEA+metformin mice. Figure A-C show the representative images of MMP stained with JC-1 of control (A1-4), DHEA (B1-4) and DHEA+metformin mice (C1-4) (Bar is 100μm). D, The red/green fluorescence intensity in MII oocytes was determined in all groups. Data are the mean±SEM; n shows the number of oocytes from 3–5mice per group. * P<0.05 versus control by one-way ANOVA, Dunnett post test.

The mitochondrial membrane potential (MMP) of oocytes was determined by staining the cells with inner membrane potential dye JC-1. JC-1 emits green fluorescence when the MMP Δψm is lower than 100mV and emits red fluorescence when the MMP is increased. The reduction of MMP, determined by the ratio of red to green fluorescence, is considered to be an initial manifestation and hallmark of mitochondrial damage[17]. We found, the mean ratio of red/green fluorescence intensity of MII oocytes from the DHEA group was significantly decreased compared to the controls (1.09±0.04 vs. 1.44±0.11). Metformin treatment (1.17±0.046) only slightly hampered the damage, with no significant difference with the control or DHEA group (Fig 5).

### DHEA treatment enhanced the oxidative stress in MII oocytes, which was reversed by metformin treatment

**Fig 6:**
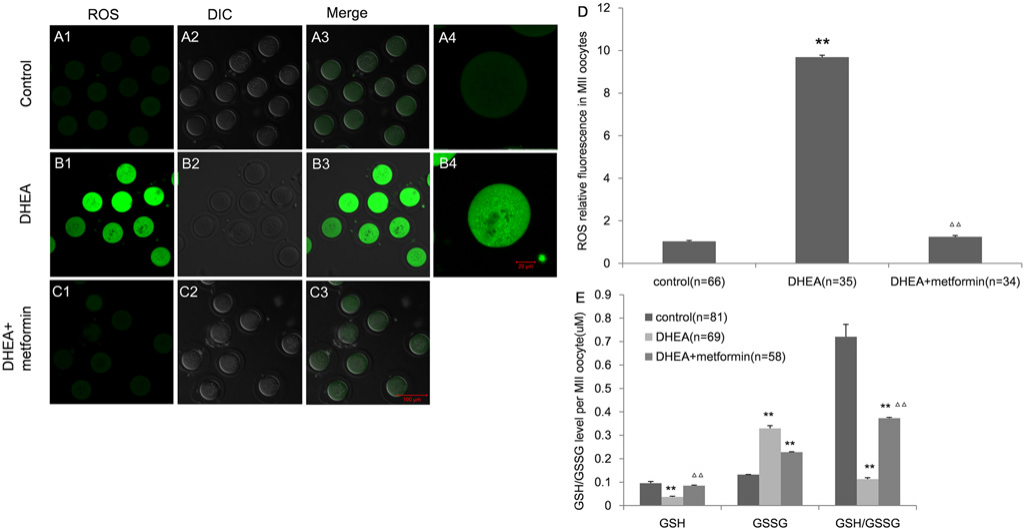
Effect of DHEA and metformin on the oxidative stress in MII oocytes. Figure A-C show representative images of the ROS in the control (A1-3), DHEA (B1-3) and DHEA+metformin group (C1-3) (Bar is 100μm). A4 is a magnified view of one oocyte from A1 and B4 is a magnified view of one oocyte from B1(Bar is 20μm). D, The DCFH-DA fluorescence intensity in the three groups. E, The GSH concentration and GSH:GSSG ratio in MII oocytes. Data are the mean±SEM; n shows the number of oocytes from 3–5mice per group. ** P<0.01 versus control, Δ Δ P<0.01 versus DHEA group by one-way ANOVA, Dunnett or Bonferroni post test.

Intracellular ROS was measured to evaluate the oxidative stress in oocytes. The DCFH-DA fluorescence intensity was significantly higher in the DHEA group than that in the control group, suggesting an increased production of ROS. The augmented ROS production was greatly reversed by metformin treatment. Representative images are shown in Fig 6A–6D.

GSH is an important antioxidant. The GSH level and the ratio of GSH to GSSG are regarded as important parameters of the antioxidant capacity of the body. As shown in Fig 6E, the GSH level and GSH/GSSG ratio were significantly decreased in the DHEA group compared with the controls (0.037±0.003 vs. 0.095±0.008 of GSH level; 0.11±0.006 vs. 0.72±0.05 of GSH/GSSG). However, when metformin was added, they were all significantly increased (0.085±0.003 of GSH level; 0.37±0.004 of GSH/GSSG). Together, these results indicated that DHEA treatment disrupted the antioxidant functions in mice and this disruption can be rescued by metformin to a certain degree.

### Spindle assembly was not influenced by DHEA in MII oocytes

**Fig 7:**
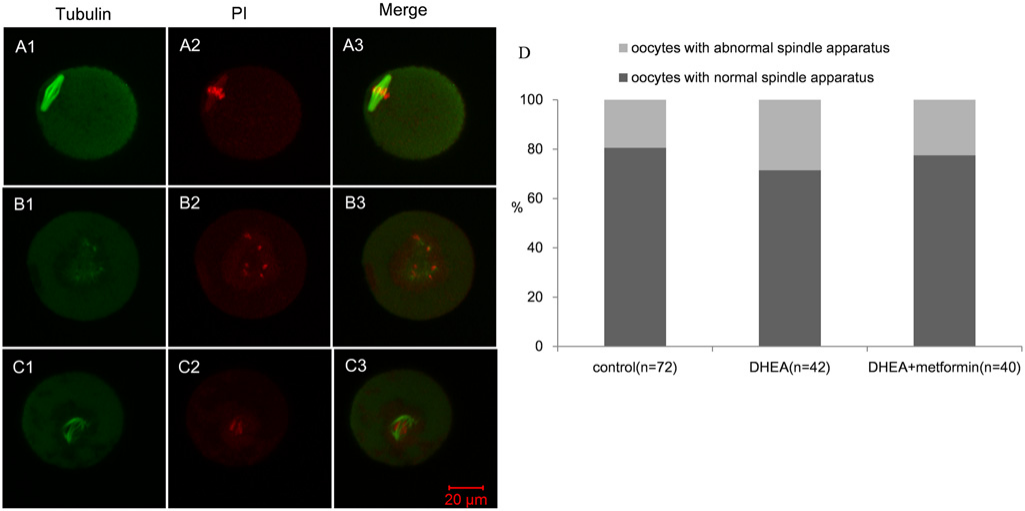
Effect of DHEA and metformin on the spindle apparatus of MII oocytes. Figure A1–3 shows the representative images of normal spindle apparatus. Abnormal spindle apparatus was characterized by dispersed spindles and chromosomes (B1–3) and abnormal spindle shapes (C1–3) (Bar is 20μm). Figure D shows the constituent ratio of oocytes with a normal and abnormal spindle apparatus in MII oocytes by chi square test.; n shows the number of oocytes from 3–5 mice per group.

Spindle and chromosome assembly of the oocytes matured in vivo was examined by immunofluorescence staining and observed under confocal microscopy. A total of 154 MII oocytes were collected for analysis. As shown in Fig 7A, oocytes with chromosomes located exclusively on the equatorial plate and a shuttle-shaped spindle structure were considered to be normal [18]. Abnormal spindle and chromosome assembly were characterized by dispersed spindle and chromosomes, abnormal spindle shapes or no spindle structure (Fig 7B and 7C). Normal and abnormal spindle ratios were calculated in the three groups. As shown in Fig 7D, 28.57% defective spindles and chromosome alignment were observed in the DHEA group, which was higher than that in the control group (19.44%) and DHEA+metformin group (22.5%). However, no statistical difference was found among them.

### DHEA treatment compromised the maturation potential of GV oocytes

**Fig 8:**
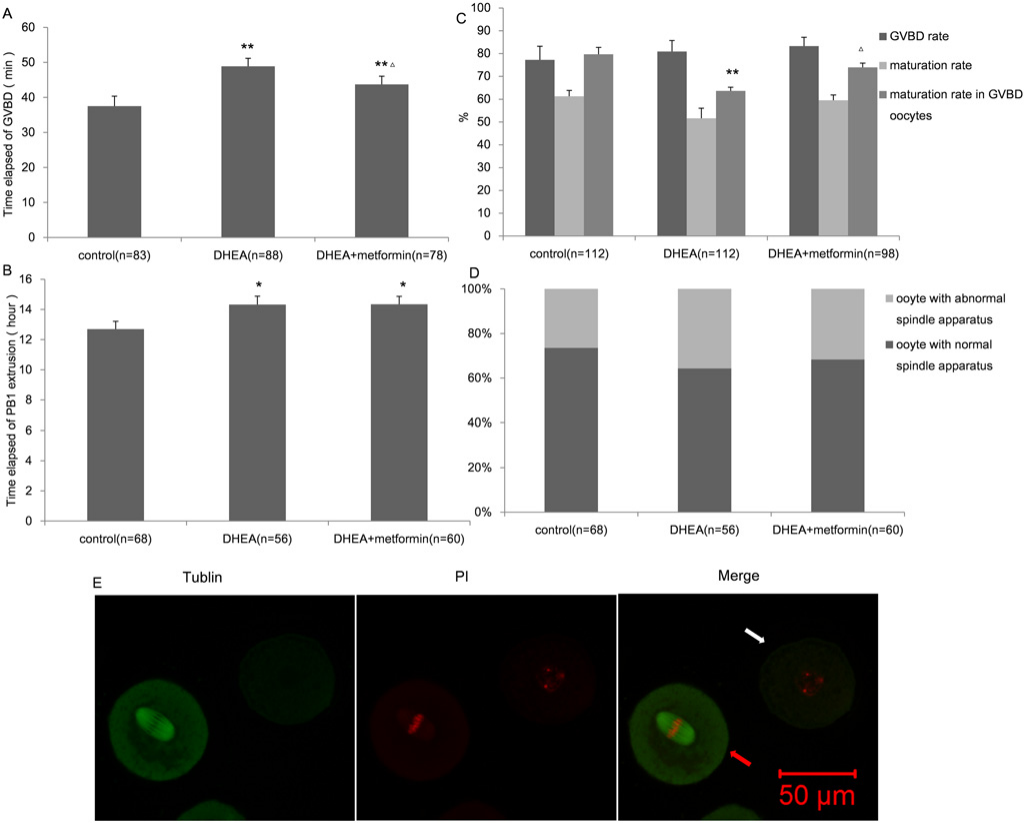
Maturation potential of GV oocytes in control, DHEA and DHEA+metformin mice. A, The time elapsed until GVBD (time elapsed between the beginning of the experiment and GVBD). B, The time elapsed until PB1 extrusion (time elapsed between the beginning of the experiment and PB1 exclusion). C, The rate of GVBD and maturation in the three groups, as well as the maturation rate of the oocytes that had accomplished GVBD. D, The percentage of oocytes with a normal or abnormal spindle apparatus; E, Representative images of normal and abnormal spindle apparatus. Normal spindle apparatus was characterized with chromosomes located exclusively on the equatorial plate and a barrel-shaped structure (red arrow). The white arrow shows an abnormal spindle apparatus with dispersed chromosomes. Data are the mean±SEM and n shows the number of oocytes from 6–8 mice per group. * P<0.05, ** P<0.01 versus control; Δ P<0.05 versus DHEA group by one-way ANOVA, Scheffe or Bonferroni post test. Statistical analysis of the normal spindle ratio was performed by chi square test.

To evaluate the maturation potential of GV oocytes, immature oocytes from the three groups were cultured in IVM medium for 24 h under the live cell station. As shown in Fig 8A and 8B, oocytes from the DHEA group demonstrated a significantly delay for their GVBD and PB1 extrusion compared to the control group (48.85±2.34 vs. 37.5±2.86 min of GVBD; 14.32±0.56 vs. 12.69±0.52 h of PB1 extrusion). Metformin administration counteract the delay of GVBD (43.71±2.34 min), but had no effect on PB1 extrusion (14.34±0.53 h). The GVBD and maturation rate of the three groups did not differ from one another, but among the oocytes that have accomplished GVBD, the maturation rate was significantly decreased in the DHEA group (Fig 8C). In addition, the oocytes that matured in vitro were further used for chromosome alignment and spindle structure evaluations. However, as shown in Fig 8D, no obvious change was found among these three groups. In general, DHEA damaged the biological process during in vitro maturation, whereas metformin rescued the harm in terms of the time elapsed of GVBD and the maturation rate in GVBD oocytes.

## Discussion

In the present study, we investigated the effects of DHEA and metformin on maternal metabolism and oocyte quality in mice. The results indicated that excessive DHEA disrupted maternal metabolism and fertility function, hampered the oocyte quality and decreased in vitro maturation and developmental potential. However, metformin treatment partially rescued these impairments and improved the oocyte quality.

In 1962, the observation of increased DHEA levels in PCOS women [19] led to the development of a PCOS animal model by injecting DHEA as an inducer of polycystic ovaries [20]. Since then, DHEA-induced murine PCOS models have been widely used. The postnatal treatment of rodents with DHEA resulted in all or most of the females exhibiting follicular cysts and representative features of human PCOS, such as acyclicity, anovulation, polycystic ovaries, and hyperandrogenism[21]. However, recent studies demonstrated controversial effect of DHEA on body composition and metabolic profiles. Some studies showed that DHEA supplementation decreased body weight and increased the serum insulin level and HOMA index[22,23,24], whereas others suggested that DHEA treatment hampered insulin sensitivity with unchanged or increased body weight[25,26,27]. However, in the studies reporting the former conclusion, DHEA was administered orally, which has been proven to reduce protein digestibility and the nutrient transformation[24]. Thus, the anti-obesity and insulin improvement functions of DHEA may be mainly due to its influence on protein digestion. Subcutaneous injection may avoid this, leading to a totally different effect on body weight and lipid metabolism. Our research showed that DHEA treatment significantly increased the body weight and fat mass of mice, with impaired glucose tolerance and dyslipidemia. The mechanism of DHEA-induced metabolic disturbance is not completely clear and might be related to the increased testosterone level transformed from DHEA. Metformin, an insulin-sensitizing drug, exerts a systemic action on the regulation of glucose metabolism by insulin. By increasing the muscle glucose uptake and decreasing hepatic glucose production, it led to fat loss and metabolic improvement in the DHEA-treated mice observed in our study.

PCOS patients are often associated with poor oocytes quality during IVF, and hyperandrogenism might be a main reason. The full developmental competence of an oocyte requires the synchronous maturation of the nucleus and cytoplasm [28]. Any dysfunction or dislocation of oocyte components could impair oocyte quality. The decreased number of total retrieved and normal MII oocytes and increased MI oocytes demonstrated a maturation and ovulation disorder during superovulation in DHEA treated mice. The spindle apparatus of MII oocytes matured in vivo and in vitro were further detected to investigate the situation of nuclear maturity. No significant difference was found between the DHEA and control group. This finding is consistent with the fact that the chromosomal constitution of PCOS patients is similar to that of controls [29,30].

Among the changes that occur during cytoplasmic maturation, the functional status of mitochondria, which are the most abundant organelles in mammalian oocytes, play a primary role. DHEA, an adrenal steroid, is known to have an anti-proliferative effect and is associated with mitochondrial function impairment. Previous studies have shown that DHEA treatment can cause neuronal mitochondrial dysfunction and lipid peroxidation in rat liver mitochondria [31,32]. In addition, DHEA also inhibits mitochondrial respiration in vitro, and incubation with DHEA led to a dose- and time-dependent decrease in cellular ATP levels [33,34]. The mitochondrion is both the main origin and attack target of ROS. In vitro and in vivo experiments have demonstrated that DHEA supplementation sensitizes leukocytes to promote oxidative stress in humans [35,36]. DHEA-treated mice also showed lipid peroxidation and decreased superoxide dismutase activity in ovarian tissues [37]. Significantly increased ROS levels and decreased GSH in our research demonstrated an obvious oxidative stress status in the oocytes. Excessive ROS can attack the mitochondria, while the impaired mitochondria, in turn, can increase the generation of ROS, resulting in oxidative damage and forming a vicious circle. The specific mechanism of mitochondria damage and oxidative stress caused by DHEA is not completely clear. D.Safiulina’s study demonstrated that DHEA inhibits mitochondrial respiration by directly acting on complex I of the respiratory chain [32]. In addition, we speculated that lipid accumulation observed in the oocytes accompanied by fat gain and lipid metabolism disturbance in DHEA-treated mice might also be one of the influence factors. The accumulation of intracellular lipids leads to high levels of free fatty acids that are subject to oxidative damage and lipid peroxides, which are ultimately detrimental to intracellular organelles, particularly mitochondria [38]. Mitochondrial function is quite important during oocyte cytoplasm maturation. The mitochondrial dysfunction induced by DHEA would inevitably cause great damage to the oocyte quality, resulting in impaired fertilization and development competence. However, because of the absence of “in vitro” treatment component of our study, it is hard to attribute the alterations in oocyte quality observed in DHEA treated mice to DHEA alone versus metabolic derangements induced by DHEA.

Although the systemic effect of metformin has been identified in PCOS patients, the local effect on oocyte maturation is not quite clear. During the process of maturation, the oocyte obtains most of the energy from cumulus cells, which metabolize glucose via glycolysis to produce pyruvate and lactate, the preferred substrates of the oocyte[39]. Metformin was reported to have the potential to stimulate the production of lactate in human granulosa cells[40] and thereby improve “nutrition” for the developing oocyte and increase the metabolic substrates for ATP production. In addition, lipids are also an important energy source for oocyte maturation, and metformin can increase the usage of lipids and β-oxidation of fatty acids, which is essential for mouse oocyte developmental competence and early embryo development [41,42]. The increased energy source in the oocyte and the enhanced catabolic processes that generate ATP lead to the increased ATP level and decreased lipid content as observed in the DHEA+metformin mice. Moreover, metformin treatment reversed the oxidative stress induced by DHEA, in terms of ROS reduction and increased antioxidant defenses in the oocytes. Previous reports have demonstrated that metformin exposure in vivo is associated with lower oxidative damage and the inhibition of chronic inflammation in the liver, which is induced by reduced superoxide production and Nrf2-dependent antioxidant response activation [41]. In addition, a therapeutic concentration of metformin normalizes mitochondrial H2O2 emission by blocking reverse electron flow without affecting forward electron flow or respiratory O2 flux in the skeletal muscle of obese rats [43].

However, it is worth noting that some previous studies demonstrated that metformin can inhibit the mitochondrial ETC complex I activity and impair mitochondrial function [44,45]. The reason of this discrepancy with our results might because the dose that used in vivo is much lower than that used in most of the in vitro models. The inhibition of mitochondrial respiration by metformin is reported to be concentration-dependent and far less sensitive than complex I-linked mitochondrial H2O2 emission [43]. In addition, the adaption and regulation of the body might also contribute to the different effect between the in vivo and in vitro studies.

As the technology of live cell stations and time lapse has just started emerging, studies of the nuclear dynamics during oocyte maturation have been limited at this point. Escrich’s study demonstrated that the time required for nuclear maturation, which is important for the resumption of meiosis and normal activation, depends on the duration of the GV stage rather than that of MI arrest (time elapsed between GVBD and PB1 extrusion)[46]. These results suggest that the duration of the GV stage is quite important in oocyte maturation during IVM. The prolonged GV stage duration and the time required for PB1 extrusion in DHEA-induced mice suggests that DHEA administration disturbs the normal process of nuclear maturation. The delay in GVBD might cause a developmental deficiency of the oocytes, leading to their inability to complete the subsequent extrusion of the first polar body.

## Conclusions

This study is the first to confirm a direct impairment of excessive androgen on oocyte quality, which can be partially rescued by metformin treatment. However, to achieve the goal of obtaining a PCOS-like mouse model, the DHEA was given in a quite high dosage, which far exceed the androgen levels in PCOS patients, makes it difficult to extrapolate to humans. Also, because of the physiological differences between mice and humans, the mouse model used in this study cannot fully represent the ture situation of PCOS patients. Nevertheless, our study provided a clue that hyperandrogenism might be a direct cause of poor oocyte quality in PCOS patients during IVF, whereas metformin pretreatment might reverse the impairment. Further studies should be conducted to confirm this effect in clinical studies and elucidate the mechanism of the improvement induced by metformin.
